# Opportunity and Ambition

**Published:** 1915-10

**Authors:** 


### Opportunity and Ambition.

We heard an eloquent sermon, recently, in which the speaker recounted some tragedies of disappointed ambition and as we applied his illustrations to our own profession, indignation arose at the men who tread on the fingers of those beneath them on the ladder which leads to success and great achievement and we thought of the terrible responsibility assumed by those who, for selfish ends or on account of personal preferences for others, stunt the development of men who with a little encouragement or, without obstacles deliberately placed in their way, might accomplish so much.

Then, as the speaker mentioned the case of an elevator boy whose ambition was to become a preacher—"without opportunity and, apparently without the slightest fitness"—and drew a sharp differentiation between ambition "to do great things" and "to be the one to do them," we realized that there were two sides to the question. A good deal of the ambition in all walks of life, is to get the most for the individual, at the least exertion; not to do the most for the people and to work as hard as one reasonably can in developing his inherent ability. Somehow, obstacles have never impressed us much. Often, the opportunities offered are not in accordance with individual fitness and preference and there is a good deal of satisfaction in feeling that one has not been boosted, although it must be admitted that the fable of sour grapes may be applied to this statement.

The speaker also emphasized the fact that it was a good deal more important to have a definite thing accomplished than to have it accomplished by any particular person. It must be admitted that the majority of the men in medicine who owe their chance to social position and personal influence, have made good. In any line of work, the same is true and, the man whom we naturally blame for giving opportunity according to personal favor, undoubtedly feels that it is right to use his acquired power in this direction. And, yet, we can't help wishing that our democratic theories were more literally applied.

